# Interleukin-12 in multimodal tumor therapies for induction of anti-tumor immunity

**DOI:** 10.1007/s12672-024-01011-2

**Published:** 2024-05-16

**Authors:** Yulian Xu, Xueli Sun, Yunguang Tong

**Affiliations:** 1 College of Life Sciences, China Jiliang University, 168 Xueyuan Street, Hangzhou, Zhejiang China; 2 College of Pharmaceutical Sciences, Zhejiang University, Hangzhou, 310058 China; 3 Omigen, Inc, Hangzhou, 310018 Zhejiang China

**Keywords:** Cytokine, IL-12, Combination therapy, Anti-tumor immunity

## Abstract

Interleukin-12 (IL-12) can be used as an immunomodulator in cancer immunotherapy. And it has demonstrated enormous potential in inhibiting tumor growth and improving the tumor microenvironment (TME) by several preclinical models. However, some disappointing results have showed in the early clinical trials when IL-12 used as a single agent for systemic cancer therapy. Combination therapy is an effective way to significantly fulfill the great potential of IL-12 as an immunomodulator. Here, we discuss the effects of IL-12 combined with traditional methods (chemotherapy, radiotherapy and surgery), targeted therapy or immunotherapy in the preclinical and clinical studies. Moreover, we summarized the potential mechanism underlying the anti-tumor effect of IL-12 in the combination strategies. And we also discussed the delivery methods and tumor-targeted modification of IL-12 and outlines future prospects for IL-12 as an immunomodulator.

## Introduction

According to data from the International Agency for Research on Cancer, cancer was responsible for 9.74 million deaths [1], making it the second leading cause of death worldwide after cardiovascular disease [2, 3]. Because of the complexity and difficulty of cancer treatment, researchers have proposed immunotherapy as an alternative to traditional methods such as surgery and chemo-radiotherapy. Immunotherapy can kill cancer cells and tumor tissue by artificially activating the immune system and harnessing its autoimmune function [4]. The development of immunotherapy represents a major milestone in cancer treatment [5]. In order for the immune system to effectively kill cancer cells, a continuous series of events must be initiated, perpetuated, and expanded, which was called the cancer immunity cycle [6]. Many cytokines involved play important roles in this cycle. Thus, researchers have investigated the influence of single factors on the overall tumor immune response, laying the foundation for subsequent studies. Such as interleukin-2 (IL-2), one of the prototypical examples of successful cytokine-based immunotherapy for cancers, has been studied for over 47 years. IL-2 is used to stimulate T cell production for enhancing anti-cancer immunity. It is approved for treatment of advanced melanoma [7, 8] and metastatic renal cancer [9, 10]. Interferon-alfa (IFN-α) is approved for the treatment of melanoma [11] and hairy cell leukemia [12]. But the low response rates and toxicity limited the number of patients who can benefit from IL-2 or IFN-γ, so it is necessary to find more effective cytokines that can effectively assist conventional treatments. Interleukin-12 (IL-12), a cytokine which has been studied as an immunomodulator frequently in recent years, showed an outstanding anti-tumor effect in preclinical studies.

In this review, we discuss the role of IL-12 as an immunomodulator in promoting anti-tumor effect briefly and the application of IL-12 in combination with other therapies in the preclinical and clinical studies.

### Biological activity of IL-12

IL-12, a member of heterodimeric cytokines family, was first discovered and characterized in 1989 by two independent groups [13, 14]. IL-12 is a heterodimeric molecule composed of an α-chain (p35 subunit) and a β-chain (p40 subunit) linked by a disulfide bridge to form the biologically active 74 kDa heterodimer [15]. The IL-12 signaling pathway was activated by the binding of IL-12 and its receptor (IL-12R), which triggers the production of IFN-γ by tyrosine kinase 2 (Tyk2), Janus kinase 2 (JAK2), and signal transducer and activator of transcription 4 (STAT4) [16, 17].

**Fig. 1:**
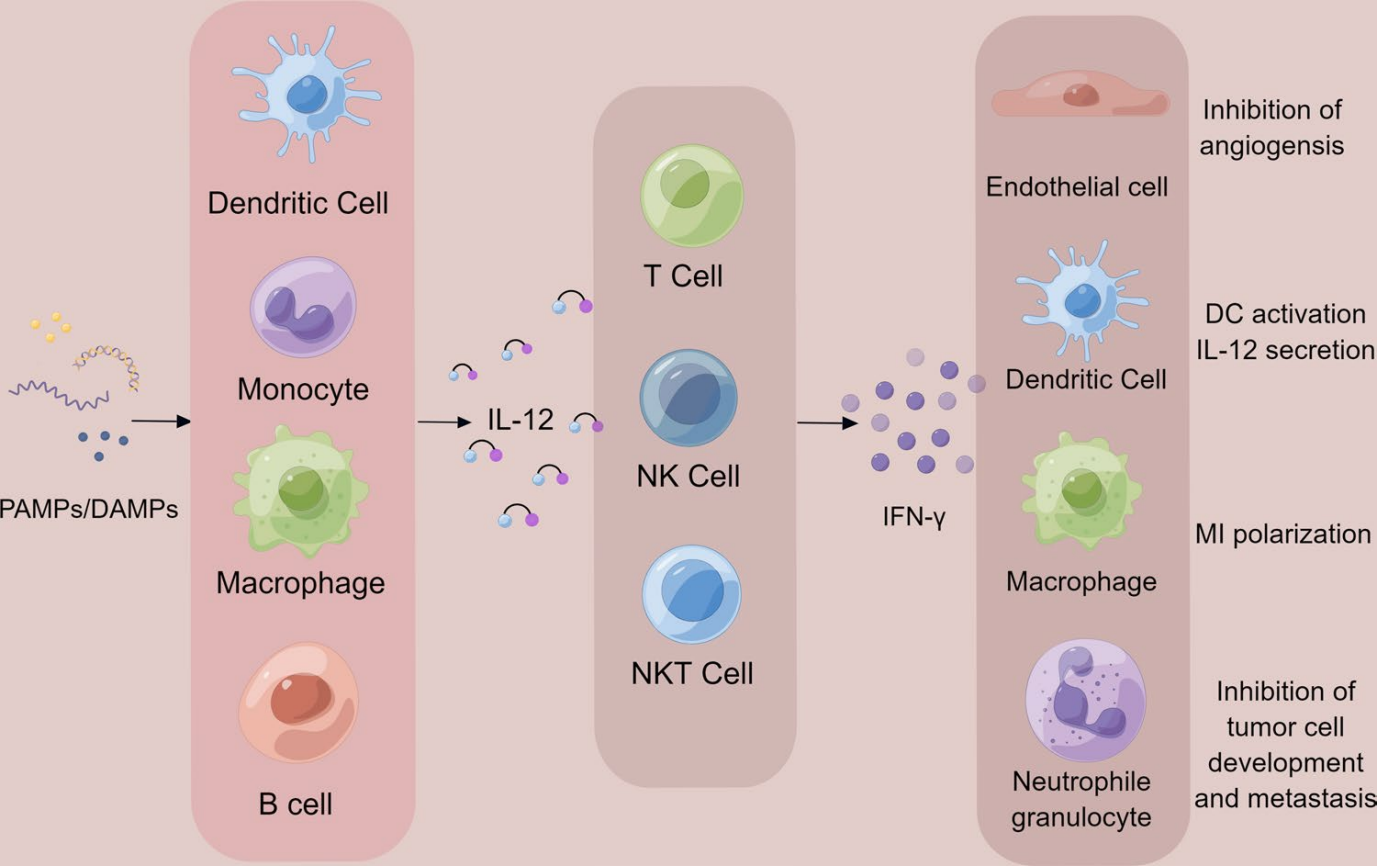
Summary of the physiological origin of IL-12 and its effects on downstream cells. The figure highlights that DCs, monocytes, macrophages and B cells will secrete IL-12 by stimulation. The major sensing cells are T cells and NK cells, and the major downstream effector molecule is IFN-γ. PAMPs/DAMPs: pathogen-associated molecular patterns/damage-associated molecular patterns

IL-12, mainly produced by dendritic cells, monocytes, macrophages and B cells, can activate many subsets of immune cells which can recognize and destroy cancer cells, and stimulate immunity during the cancer immunity cycle. The anti-tumor activity of IL-12 is reflected in its ability to induce effector cells, including NK, NKT and T cells, to produce IFN-γ, TNF and other cytokines. The major downstream effector molecule of IL-12 is IFN-γ, which can prevent the proliferation and promote the apoptosis of tumor cells, inhibit angiogenesis, and stimulate the innate and adaptive immune systems. IFN-γ can also stimulate phagocytes and promote the maturation of DCs to produce IL-12 [18, 19]. Figure 1 provides a graphical summary of the functions of IL-12 and the cells it affects, mainly focusing on the downstream effects of IL-12 mediated by IFN-γ. IL-12 can enhance the cytotoxic effects mediated by NK cells and T cells, especially CD8^+^ T cells, in the immunosuppressive microenvironment and participate in cell proliferation and CD4^+^ Th1 cell adhesion. IL-12 is a promising candidate for the anti-tumor treatment due to the impressive immunomodulatory effects based on the preclinical and clinical studies [20, 21].

### The development of IL-12 based therapies

Since IL-12 was first studied [22], there have been much research on the effect of IL-12 against a variety of transplantable mouse tumors (such as melanoma [23], colon tumors [24, 25], and mammary tumors [26, 27]). However, some side effects were observed in the preclinical studies. Such as hematologic toxicities including anemia, lymphopenia, neutropenia, muscle and hepatic toxicities [28]. Moreover, hypoproteinemia, hypophosphatemia, hypocalcemia, enlargement of lymph nodes, splenomegaly, and bone marrow hyperplasia were also observed in squirrel monkeys treat with recombinant human IL-12 [29]. These side effects may be provoked by IFN-γ and TNF-α production stimulated by IL-12.

As for the early clinical trials, in the mid-1990s showed that systemic administration of IL-12 caused dose-limiting toxicity. In a phase II trial, the maximum dose of 0.5 μg/kg/day resulted in serious side effects in 12 of 17 patients and death in 2 patients (40 patients) [30, 31]. But the dose of 0.5 μg/kg/day was the maximal tolerated dose determined in the phase I, the explanation for the different tolerability in phase I versus phase II trial was a change in the dosing schedule. The serious incident resulted in the immediate halting of all trials with IL-12 by the FDA. And finally, after several months of suspension, clinical trials were resumed in several centers. Hematologic toxicity observed most commonly was neutropenia and thrombocytopenia, and hepatic dysfunction manifested in transient (dose-dependent) increase in transaminases, hyperbilirubinemia, and hypoalbuminemia [32–36]. Some patients experienced inflammation in mucus membranes (oral mucositis, stomatitis, or colitis) [34].

Current research on IL-12 is mainly focused on avoiding or minimizing these side effects to fully exploit the ability of IL-12 [37–39]. Combination therapy has been the mainstream trend against the cancer. It can enhance efficacy compared to the monotherapy because it targets key pathways in a characteristically synergistic or an additive manner. And it potentially reduces drug resistance as well as providing therapeutic anti-tumor benefits simultaneously. The combination trials of IL-12 and other treatments are underway, involving in the delivery methods and modification of IL-12, which significantly improves the TME and achieves better therapeutic effects. For example, by combining anti-programmed cell death 1 ligand 1 (PD-L1) with mRNA encoding IL-12, the mechanism underlying the anti-tumor activity of IL-12 mRNA was confirmed [40]. The results demonstrate the potential for intratumorally delivered IL-12 mRNA to promote Th1 transformation of the TME and robust anti-tumor immunity. It also provides a new therapeutic approach for cancer treatment, and combining low-dose IL-12 with other cytokines and therapeutics can elicit a synergistic and recurrent anti-tumor immune response.

Here, we summarize how IL-12 has been used in combination with traditional methods (chemotherapy, radiotherapy and surgery), targeted therapy, and immunotherapy in preclinical studies and a small number of clinical trials. Finally, we discuss the current challenges related to the use of IL-12 and propose promising solutions.

## IL-12 therapy in combination with traditional methods

The traditional methods of cancer treatment mainly include chemotherapy, radiotherapy and surgery. But these conventional treatments always accompanied by lots of critical side effects, so it’s necessary to find new and efficient therapies to avoid or alleviate side effects. Recently, the combination of traditional methods and IL-12 has attracted much interest, and there are some valuable results achieved in the exploration of combination therapy.

### The combination of chemotherapy and IL-12

Chemotherapy is one of the most common treatments used to inhibit tumor growth. Conventional chemotherapeutic drugs exert anti-tumor effects by inhibiting cancer cells via induction of cell cycle arrest and/or cellular apoptosis [41]. However, the patients treated chemotherapy often suffer serious side effects, such as vomiting, nausea, anemia, and fatigue [42], due to the toxic effects on normal cells. In addition, tumor cells often become resistant to chemotherapeutic drugs, which leads to tumor recurrence and further disease progression [43, 44]. The combination of IL-12 and chemotherapeutic drugs showed an outstanding anti-tumor effect as showed below.

The combination strategy involves IL-12 and paclitaxel (PTX)-coloaded nanoparticles containing 10 μg/kg IL-12 and 4 mg/kg PTX (IL-12/PTX@TSNP_pH_) [45]. Compared with the saline group and the other single-agent groups, the tumor inhibition rate in the IL-12/PTX@TSNP_pH_ group increased to 84.3%, and the number of metastatic nodules decreased on the surface of lung tissues (47 ± 4 in the saline group and 10 ± 2 in the IL-12/PTX@TSNP_pH_ group). The combination therapy significantly repressed metastasis and prolonged the overall survival of mice without any significant toxic side effects. The combination of IL-12 and PTX induced the release of IFN-γ from T lymphocytes and NK cells, selectively inhibited regulatory T cells (Tregs), induced tumor-associated macrophage polarization toward the M1 type, and improved the immunosuppressive tumor microenvironment and killed tumor cells [45]. The combination of vincristine (VB) and IL-12-expressing oncolytic herpes simplex virus vectors (NV1042) exhibited additive or synergistic effects on prostate cancer cell lines [46]. The combination of NV1042 and VB reduced tumor size significantly than the other treatments (NV1042/VB: 487 ± 175 mm^3^; NV1042: 1087 ± 254 mm^3^; VB: 1623 ± 611 mm^3^). The potential mechanism of the combination may due to the significant reduction in the number of CD31^+^ cells, and it may exert anti-tumor and antiangiogenic effects. And the combination of IL-12 and VB was nontoxic in prostate cancer models. The combination IL-12 and oxaliplatin (OXP) [47], which maintained continuous and low-level controlled expression of IL-12 with reduced toxicity, was developed as a potential strategy for liver metastases. The combination of IL-12 and OXP induced a more immunogenic phenotype of the TME, with an increased ratio of CD8^+^ T-regulated cells and a decrease in the number of myeloid-derived suppressor cells, then prevented liver metastases of MC38Luc1 tumors.

In a phase I clinical trial [48], the IL-12 plasmid (phIL-12) formulated with the lipopolymer PEG-PEI-cholesterol (PPC), in combination with carboplatin/docetaxel were explored in women with platinum-sensitive ovarian cancer. Twelve patients were evaluable for response after the combination treatment among thirteen patients, with 17% complete response. The median progression-free survival and overall survival for all treatment groups were 8.8 months and 16.6 months respectively. The increased doses of phIL-12/PPC in conjunction with carboplatin and docetaxel were safe and well tolerated. This combination strategy offers a unique advantage over conventional cytotoxic therapies due to its safety when used long-term treatment.

In addition, there are also some ongoing trials about the combination of IL-12 and chemotherapeutic drugs for the treatment of cancer (Table 1). Such as rhIL-12/filgrastim/etoposide/ifosfamide and IL-12/carboplatin/paclitaxel. Although the preclinical findings above provided evidence for the efficacy of IL-12 combined with chemotherapy, there are few relatively reliable clinical results to confirm the clinical safety and efficacy of IL-12 combined with chemotherapeutic drugs in the treatment of cancer, so the further research is needed to confirm the credibility and feasibility of this combination therapy.

**Table 1:** Clinical trials of IL-12 combined with chemotherapy, radiotherapy and targeted therapy

IL-12	Combination regimen	Status	Phase	Condition	CT number
IL12-FHAB	Filgrastim, etoposide, ifosfamide	Completed	II	•Lymphoma	NCT00003575
GEN-1	Carboplatin, Paclitaxel	Active, not recruiting	I and II	•Epithelial ovarian •Fallopian Tube	NCT03393884
GEN-1	Standard Neoadjuvant Chemotherapy	Completed	I	•Epithelial ovarian cancer •Fallopian tube cancer	NCT02480374
GEN-1	Pegylated Liposomal Doxorubicin Hydrochloride	Completed	I	•Ovarian clear cell •Cystadenocarcinoma	NCT01489371
NHS-IL12	Bintrafusp Alfa, Entinostat	Recruiting	I and II	•Oropharyngeal cancer •Neck cancer •Human Papillomavirus	NCT04708470
NHS-IL12	Bintrafusp Alfa, SBRT	Recruiting	I	•Urothelial cancer •Bladder cancer •Genitourinary cancer	NCT04235777
M9241	SBRT	Recruiting	I	•Cancer of prostate	NCT05361798
PCX-12	SBRT	Not yet recruiting	I	•Pancreatic •Adenocarcinoma	NCT06217666
NHS-IL12	Bintrafusp Alfa, RT	Withdrawn	I	•Hormone receptor positive breast •Adenocarcinoma •Metastatic breast carcinoma	NCT04756505
NHS-IL12	CV301, MSB0011359C, N-803	Completed	II	•Small bowel cancers •Colorectal cancers	NCT04491955
IL12-L19L19		Recruiting	I	•Advanced solid tumor •Metastatic solid tumor	NCT04471987
NHS-IL12	Bintrafusp Alfa(M7824)	Recruiting	I and II	•Kaposi sarcoma	NCT04303117
IL-12	Rituximab	Completed	I	•Lymphoma	NCT00004260
rIL-12	ABI-007, carboplatin, trastuzumab	Completed	I	•Bone metastases •Gastrinoma •Glucagonoma	NCT00004074

### The combination of radiotherapy and IL-12

The radiotherapy (RT) is a primary treatment method of localized tumors and regional lymph nodes in a curative setting [49]. RT can inhibit the growth and division of tumor cells and induce cell death through DNA damage. Even RT has been used in the treatment of many cancers widely, but it can lead to genetic mutations [50, 51], secondary malignancies [52, 53] and many other side effects [54]. Due to the abscopal effect induced by RT might be dependent on the activation of immune system [49], the combination of IL-12 and RT has been proposed and practiced.

The combination of RT and IL-12 (RT/IL-12) [55], induced dramatic tumor regression in animals bearing large subcutaneous or orthotopic HCC, as well as systemic effect against distant tumor, and prolonged survival significantly. RT/IL-12 group led to sustained tumor regression and suppression in most animals (CR in 4 of 10 mice), with the mean tumor volume being 20 ± 11 mm^3^ on day 35, which was dramatically smaller than that of either of the monotherapy group (IL-12 group: 415 ± 78 mm^3^; RT group: 375 ± 47 mm^3^; untreated group: 1598 ± 151 mm^3^). The combination of IL-12 microsphere (MS) and stereotactic body radiation therapy (SBRT), induced marked tumor reduction and cured in multiple preclinical mouse models of pancreatic ductal adenocarcinoma (PDA) [56]. IL-12 MS/SBRT group showed a robust anti-tumor effect in recalcitrant PDA tumors with the production of intratumoral IFN-γ and activation of CD8^+^ T cell. Besides, it improved the immunosuppressive TME, reduced the densities of myeloid suppressor cells and eliminated established liver metastases. Moreover, in a treatment of murine sarcoma [57], IL-12 was regarded as a radiosensitizer with radiosensitizing effect, but the underlying mechanisms of radiosensitization remain to be elucidated. Besides, the combination therapy of IL12 and RT showed superior anti-tumor effect in the mice bearing LLC tumor models [58], the murine hepatic cancer (HCa-I) model [19] and murine prostate cancer model [59].

Currently, there are a few ongoing clinical trials (Table 1) in the National Clinical Trials Network about the combination of IL-12 and RT, but most of them are on phase I, so there still is a long way to figure out the clinical effect.

### The combination of surgery and IL-12

Surgery is chosen to treat the solid tumors by most cancer patients in early stage, it can reduce the tumor burden and resolve the pathological changes caused by the local compression of the tumor tissue. It reduces the chance of tumor cells spreading and creates more favorable conditions for further treatment. However, surgery alone does not prevent tumor recurrence and/or metastasis, and patients with a high risk of recurrence receive adjuvant therapies after surgical resection. In view of the limited clinical effects of IL-12 as a monotherapy, some researchers have focused on exploring the feasibility of IL-12 as an adjuvant therapy in combined with surgery.

Localized injection of IL-12 prior to surgery has the potential to reduce recurrence rates and/or eliminate occult metastases by inducing systemic tumor-specific immunity [60, 61]. In the highly metastatic 4T1 breast cancer model [61], a group of mice received intratumoral injections of chitosan/IL-12 (CS/IL-12) prior to tumor resection surgery. About two-thirds of mice got complete clearance of lung metastases and long-term survival. In contrast, all mice treated with surgery alone died within 38 days, and mice treated with IL-12 only had a median survival of 46 days. And the overall survival of CS/IL-12 + surgery group was increased from 0 to 65%, with 67% of the mice achieving long-term tumor-free survival (IL-12 only: 24%, CS only: 0%).

In a treatment [62] of merkel cell carcinoma (MCC) by intratumoral delivery of plasmid IL12 via electroporation (pIL-12 + EP), 3 patients with locoregional MCC and 12 patients with metastatic MCC were treated with one or four cycles of pIL-12 + EP respectively. 2 patients with locoregional MCC were treated with definitive surgery after pIL-12 + EP, and there was no recurrence at 44+ and 75+ months after surgery. The overall response rate of the metastatic MCC group was 25% (3/12). Of the 10 measurable untreated lesions, 3 had distal regression. In addition, 2 patients experienced clinical responses for 16 and 55+ months, respectively. Serum IL-12 levels were not measured, but treatment was well tolerated and no serious adverse events were observed.

Besides, in the model of residual tumor after transplantation in mice by mouse sarcoma cells S180 or human renal carcinoma cell line KCC853 [63], mice inoculated with tumors were operated to partially remove tumor tissue to establish a postoperative residual tumor model. This model simulates clinical situations which tumor cells are not completely eliminated or small tumor metastases are present before surgery. IL-12 was injected to observe its effect on residual tumors or metastatic microtumors. The results showed that postoperative administration of IL-12 can significantly inhibit the growth and metastasis of residual tumor, improve the postoperative tumor-free rate, and solve the problem of tumor recurrence caused by residual tumor growth and metastasis.

IL-12 plays an adjuvant role in the combination therapy of IL-12 and surgery. The results above demonstrated the combination can effectively create a favorable condition before surgery and inhibit tumor recurrence after the surgery. But there are few relevant clinical trial data to confirm, so it is necessary to conduct more studies.

## IL-12 therapy in combination with targeted therapy

Over the past 25 years, targeted therapy has been one of the most popular topics of cancer research, it can stop cancer cell growth by interfering with specific molecules needed for tumor growth [64]. Targeted therapy has changed the standard of treatment for several malignancies [65], with the goal of increasing patients’ complete and lasting clinical response rate. The studies of targeting VEGF/VEGFR2 and targeting EGFR/HER2 in combination with IL-12 showed that a stronger anti-tumor effect.

### Targeting VEGF/VEGFR2 in combination with IL-12 therapy

The vascular endothelial growth factor (VEGF)/VEGF receptor 2 (VEGFR2) signaling pathway is one of the most important pathways for the tumor angiogenesis [66]. The studies have showed the inhibition of this pathway can promote vascular normalization, increase the intra-tumor infiltration of lymphocytes, and decrease the number and function of inhibitory immune cell phenotypes. IL-12 can regulate T-cell-mediated anti-tumor immunity but can also stimulate downstream cellular secretion of IFN-γ, which can inhibit angiogenesis, tumor growth and metastasis.

The combination of antiangiogenic agents and IL-12 can inhibit tumor vascular growth, improve the TME and enhance the immune response against tumors. For instance, the antivascular drug ABRaA-vascular endothelial growth Factor subtype 121 (VEGF_121_) in combination with interleukin IL-12 gene therapy inhibited the growth of B16-F10 melanoma [67]. The results showed that ABRaA-VEGF_121_ combined with IL-12 was more effective in inhibiting tumor growth than either group, and 20% of the mice were completely cured. In addition, the number of microvessels in the tumor was reduced after the combination therapy, providing strong experimental support for the combined application of anti-vascular growth drugs and IL-12 in cancer treatment.

The ability of IL-12 combined with angiostatin (Angio) [68] to treat glioblastoma (GBM) was assessed in two tumor models. In this study, Angio (an antiangiogenic polypeptide) and IL-12 were delivered by an oncolytic virus. The antiangiogenic activity of the combination therapy was stronger than that of Angio or IL-12 alone, and the combination of mouse Angio (mAngio) and mIL-12 further inhibited tube formation in both two models. In the MGG4 cell model, the median survival time of mice receiving combination therapy was 136 days (PBS: 98 days; mAngio: 113 days; mIL-12: 112 days). The combination of IL-12 and Angio decreased angiogenesis and VEGF expression significantly, prevented tumor growth and increased viral lysis of tumor cells.

In general, these studies provide important preclinical data for the use of combination therapy involving antiangiogenic agents and IL-12. The tumor regression and anti-angiogenesis was observed in the groups treated with both IL-12 and antiangiogenic agents. At the same time, IL-12 can change the TME by anti-angiogenesis and improve the immunosuppressive TME, and then achieve better effects in the treatment.

### Targeting EGFR/HER2 in combination with IL-12 therapy

Epidermal growth factor receptor (EGFR) and human epidermal growth factor receptor 2 (HER2) belong to the ErbB family of tyrosine kinase receptors. EGFR signaling cascades are key regulators of cell proliferation, differentiation, division, survival, and cancer development. HER2/neu is thought to be required for initiating and maintaining the growth and progression of tumors overexpressing HER2/neu, which is an attractive therapeutic target due to the characteristics of HER2/neu expression in breast cancer [69].

Cetuximab [70] is a recombinant chimeric human mouse immunoglobulin G1 antibody that binds to the extracellular domain of EGFR with a higher affinity than either of its endogenous ligands. It can inhibit the cell cycle, tumor progression, neovascularization, invasion, and metastasis. The combination of cetuximab and IL-12 increased NK cell-mediated tumor cell killing in a mouse model of squamous cell carcinoma of the head and neck (SCCHN) [71]. Moreover, the combined effect was also proved in the patients with unresectable primary or recurrent SCCHN in a phase I/II clinical trial (NCT01468896) [72]. When used in the combination therapy, the maximum tolerated dose of IL-12 was 0.3 μg/kg, and the combination of IL-12 with cetuximab was well tolerated. 69% of patients had stable disease without partial remission, and 48% of patients exhibited prolonged PFS (average of 6.5 months) after the treatment of IL-12 and cetuximab. Therefore, low-dose IL-12 combined with cetuximab is safe in SCCHN patients and may serve as an important primer to optimize the innate immune system’s ability to stimulate an adaptive anti-tumor immune response in these patients.

Trastuzumab is a humanized monoclonal antibody against HER2/neu and represents a possible treatment for metastatic breast cancer with high expression of HER2/neu. In a combination study of IL-12 and trastuzumab [73], IL-12 enhanced the FcγR-dependent immune response through IFN-γ produced by NK cells, thereby enhancing the anti-tumor effect of trastuzumab. Compared with other groups, treatment with IL-12 and 4D5 significantly inhibited the growth of colorectal adenocarcinoma constructed in BALB/c mice expressing human HER2 (CT-26 HER2/neu) and reduced the tumor volume by approximately 70%. The mice that treated with combination therapy produced significantly higher of IFN-γ and CCL5, and lower level of IL-8 in the serum.

According to the experiments described above, the anti-tumor effects mainly depend on NK cells activated by the combination of IL-12 and targeted drugs (cetuximab and trastuzumab). IL-12 can be used at a lower dose and exhibits better efficacy when in combination with targeted therapies. In general, the studies provide important preclinical/clinical data regarding the use of IL-12 combined in targeted therapy. Moreover, numerous clinical trials of the combination are being conducted to further evaluate the clinical safety and efficacy (Table 1).

## IL-12 therapy in combination with immunotherapy

Recent studies have highlighted the feasibility of using immunotherapy that try to enhance host immune responses to tumors, and the immunotherapy has showed a powerful anti-tumor effect in many studies. Broadly defined, immunotherapy refers to the stimulation of the immune system by vaccines, cytokines, antibody/immune checkpoint inhibitors (ICIs), or immune cells themselves to fight cancer cells [74]. Immunotherapy harnesses the memory of the immune system to target tumor cells to achieve a durable treatment response and is thus a more specific and less toxic treatment options for cancer patients. IL-12 is a potent immunomodulator and may have a stronger anti-tumor effect when used in combination with immunotherapies, such as immune checkpoint inhibitors, CAR-T cells and other cytokines.

### Immune checkpoint inhibitors in combination with IL-12 therapy

Immune checkpoints are pathways with inhibitory or stimulatory properties that maintain self-tolerance and promote the immune response [75]. Immune checkpoint inhibitors (ICIs) are molecules that block these pathways to enhance host anti-tumor immunity. PD-1/PD-L1 inhibitors, the most compelling ICIs, are currently used as first-line treatments in many cancers. Which significantly improve survival in patients with advanced malignancies compared to chemotherapy.

The combination of a tumor-targeting mouse IL-12 fusion protein (NHS-muIL12) and the anti-PD-L1 antibody avelumab showed a stronger anti-tumor effect in the treatment of mouse breast cancer (EMT-6) and colon cancer (MC38) [76]. The combination of NHS-muIL12 and averumab, induced tumor regression via enhancing the infiltration of cytotoxic NK and CD8^+^ T cells. In the EMT-6 cell model, 10 μg NHS-muIL12 combined with 200 μg avelumab significantly reduced the mean volume of tumors (179.1 ± 86.5 mm^3^ reduction at 8 days; 10 μg NHS-muIL12 only: 32.2 ± 51.6 mm^3^). In the MC38 cell model, the combination of avelumab and NHS-muIL12 (2 μg) therapy prolonged the survival of MC38-bearing hormonal mice, and the median survival of these mice (35.5 days) was longer than that of the mice in the monotherapy group (avelumab: 23.5 days; 2 μg NHS-muIL12: 28 days).

The combination of IL-12 and anti-PD-L1 enhanced the effect of mIL12-mRNA in inducing anti-tumor immunity, even in the patients who is resistant to PD-L1 inhibitor [40].The human IL-12 mRNA (MEDI1191) induced dose-dependent IL-12 production, IFN-γ expression, and TH1 TME transformation in *ex-vivo* patient tumor model. Intratumoral MEDI1191 is currently being evaluated in combination with duvalumab in a phase I trial (anti-PD-L1, NCT03946800) in patients with solid tumors.

IL-12 was combined with the PD-1 inhibitor nivolumab for the treatment of recurrent glioblastoma in a phase I clinical trial (NCT03636477) [77]. The immune activation in the combination group was induced by the increase of IFN-γ secretion to tumor. Although some adverse events (AEs) occurred in the trial, the AEs were predictable and dose-dependent and could be rapidly reversed by stopping the treatment, and there were no drug-related deaths. The IL-12 plasmid combined with pembrolizumab for immunologically quiescent melanoma [78] in a phase II clinical trial (NCT02493361), showed that the combination was well tolerated in patients with low percentages of checkpoint-positive cytotoxic lymphocytes (cpCTLs), no new or unexpected toxicities were observed. Patients had a 41% objective response rate (ORR) with a 36% complete response (CR) rate. The potential mechanism of the combination therapy is the increasing of immune infiltration and maintaining of the IL-12/IFN-γ feed-forward cycle, driving the generation of intratumoral cross-presenting dendritic cell subsets with an increased TILs and emerging T-cell receptor clones and ultimately promoting systemic cellular immune responses.

In addition, CTLA-4 is also a immune checkpoint that is being investigated as a target for combination therapy. CTLA-4 is a member of a family of immunoglobulin-related receptors that are responsible for various aspects of T-cell immune regulation [79]. The tumor growth in the combination of IL-12 and CTLA-4 blockade group were complete remission in most mice, compared with the IL-12 or anti–CTLA-4 conferred only a minor or no survival advantage respectively. The combination of IL-12 and CTLA-4 blockade acts predominantly on CD4^+^ T cells, causing a drastic decrease in the number of FoxP3^+^ T reg cells and an increase in the number of effector T (Teff) cells [80]. The study provided evidence for the combination strategy of IL-12, but more research is needed to confirm the effectiveness of this combination in clinical.

### CAR-T cells in combination with IL-12 therapy

Chimeric antigen receptor T (CAR-T) cells are T cells that are genetically engineered to express antigen-specific, non-MHC-restricted receptors fused to the transmembrane domain and intracellular signaling domain by a single-chain variable fragment (scFv) of an antibody [81–83]. CAR was designed to recognize tumor cells in the body and induce the release a wide range of effectors that can kill tumor cells with high efficiency to treat malignant tumors. CAR-T cell therapy is emerging as one of the most promising advances in cancer immunotherapy [81]. IL-12 can be used in combination with CAR-T cells as an immunomodulator to improve the TME, alleviate the inhibitory effects of CAR-T cells and increase activated T-cell infiltration, thus effectively elevating the anti-tumor activity of CAR-T cells [84, 85].

The combination of IL-12 (partially fused with Fc of mouse IgG3) and CAR-T-cell therapy in a preclinical study of GBM [86], led to complete eradication of established gliomas in a genetically identical mouse model. The local delivery of IL-12 boosted the anti-tumor activity of CAR-T cells, which fails to control the large established gliomas. The tumor volume was effectively controlled and the survival rate increased to 70% approximately in the combination group, compared with other groups (CAR-T/IL-12 only: 30–40%). The combination of CAR-T cells and local IL-12 also promoted the effect of CAR-T cells against aggressive and immunogenic B16 cells. The efficacy of carcinoembryonic antigen (CEA)-specific CAR-T cells combined with recombinant human IL‐12 (rhIL‐12) [87] has been explored in several types of solid tumor models with high CEA expression, such as the colorectal cancer cell line HT-29, pancreatic cancer cell line AsPC-1, and gastric cancer cell line MGC803. These data demonstrate that combined IL-12 and CAR-T cell therapy promotes an effective and persistent anti-tumor response, even in the context of a poorly immunogenic model. The rhIL‐12 effectively activated CEA‐CAR T cells and increased the cytotoxic activity of CEA‐CAR T cells against CEA-positive cancer cells. The combination of IL-12 with CAR-T cell therapy for the treatment of ovarian cancer [88] and thymoma tumor [89] confirmed that IL-12 improved the TME and increased the survival rate and persistence of T cells in vitro and in vivo, exerting a stronger anti-tumor effect, these studied provided a good theoretical reference for the application of IL-12 in combination with CAR-T.

### Cytokines in combination with IL-12 therapy

Cytokines are a class of proteins or small molecular peptides that can transmit information between cells and have immunomodulatory and effector functions. They are essential components of the TME and participate in the induction and effector phases of all immune and inflammatory responses. Cytokines have been assigned to various family groups based on the structural homologies of their receptors, and each family have various and different effect in the anti-tumor immunity. Due to the complexity of the cytokine network, the combination of IL-12 and other different cytokines is a promising strategy in the treatment of cancer by the cytokine synergy. Recently, some preclinical trials have been carried out using the combination strategy of IL-12 and other cytokines, such as IL-7, IL-18 and IL-2.

Interleukin-7 (IL-7), a member of the IL-2 superfamily, binds to its receptor via a common γ-chain subunit, leads to phosphorylation of tyrosine residues, activates downstream signaling pathways [90, 91], as well as inducing proliferation of the B and T cells. The combination of IL-12 and IL-7 may exert a long-term anti-tumor effect and it is a potential combination strategy to fulfil the anti-tumor effect of IL-12 effectively. IL-12 was delivered combined with IL-7 by a tumor-selective oncolytic vaccinia virus into tumor-immunocompetent mice [92], anti-tumor activity was observed in all three models tested, with 92.9% tumor growth inhibition in a B16-F10 melanoma model, 53.3% in a CT26.WT colon carcinoma model, and 53.3% in a LLC lung carcinoma model. Moreover, mice with complete tumor elimination were able to resist reactivation of the same tumor cells, suggesting that mice treated with the combination of IL-7 and IL-12 developed long-term tumor-specific immune memory. Another study also demonstrated that the dual expression of IL-7 and IL-12 in tumors increased the number of activated CD8^+^ T cells in tumors with poor immunogenicity compared with IL-12 expression alone [93]. Three of eleven LLC tumor model mice achieved complete regression (CR) in the combination group compared to none in the other groups (PBS, IL-7 or IL-12 alone). The combination of IL-12 and IL-7 enhanced the body’s immune response and activated an inflammatory state in previously less immunogenic tumors, contributing to complete regression of tumors and elimination of distant tumor deposits without toxicities.

Interleukin 18 (IL-18) is mainly expressed by macrophages and is a member of the IL-1 cytokine superfamily [94], which plays an important role in the inflammatory process. The exploration of IL-12 and IL-18 alone or combined in vitro on the function and receptor properties of NK cells and their subsets in patients with metastatic melanoma and healthy subjects as controls [95]. Compared with IL-12 or IL-18 alone, the combination group had significant effects on NK cytotoxicity, IFN-γ production and CD25 receptor expression in NK cells in melanoma. IL-18 acts synergistically with IL-12 to promote cytotoxicity and IFN-γ production from NK and T cells and is also involved in NK cell priming and the interaction between DCs and NK cells. In addition, Katarina M [96] studied the in vitro effects of IL-12 combined with interleukin-2 (IL-2), which has been used for decades in the treatment of melanoma, on the function and receptor expression of NK cells and their subsets. Peripheral blood mononuclear cells from 27 healthy controls and 35 patients with metastatic melanoma were stimulated in vitro with groups of monotherapy or combination therapy for functional and phenotypic analysis. Compared with monotherapy, the combination of IL-2 and IL-12 had significantly greater effects in increasing NK cell toxicity and the expression of the degranulation marker CD107a on NK cells. NK cell activity is impaired in advanced melanoma [97, 98], so the combination of IL-2 and IL-12 may be a promising combination strategy in the advanced melanoma treatment by improving NK cell function.

To maintain IFN-γ induction by recombinant human interleukin-12 (rhIL-12) and enhance its activity against melanoma and renal cell cancer, rhIL-12 was combined with IL-2 in a phase I dose escalation study [99]. 28 patients were enrolled onto the study, and the maximum-tolerated dose of rhIL-12 was 500 ng/kg. In the combination group, IL-2 significantly augmented the production of IFN-γ and IFN-γ-inducible protein-10 which were induced by IL-12, led to a three-fold expansion of NK cells. And there was one major clinical response (partial response) as well as two pathologic responses. In the combination therapy of IL-12 and IL-2, IL-2 can restore and maintain immune activation by IL-12, and has clinical activity.

Inspired by these findings, many preclinical/clinical studies are underway to evaluate the safety and efficacy of IL-12 in combination with other immunotherapies (Table 2).

**Table 2:** Clinical trials of IL-12 combined with immunotherapy

IL-12	Combination regimen	Status	Phase	Condition	CT number
SON-1010	Atezolizumab	Not yet recruiting	I and II	•Advanced solid tumor •Platinum-resistant ovarian cancer	NCT05756907
IL-12 Gene	Atezolizumab	Recruiting	I	•Non-small cell lung cancer	NCT04911166
MEDI9253	Durvalumab	Active, not recruiting	I	•Solid Tumors	NCT04613492
IL-12 Plasmids	MEDI0457, Durvalumab	Active, not recruiting	II	•Human Papillomavirus-16 Positive •Human Papillomavirus-18 Positive **•**Metastatic malignant neoplasm	NCT03439085
Edodekin alfa	Pembrolizumab	Recruiting	I	•Metastatic Malignant Solid Neoplasm •Unresectable malignant solid neoplasm	NCT03030378
M9241	Avelumab	Terminated	I	•Advanced Solid Tumors	NCT02994953
Recombinant IL-12	NA17.A2 Peptide Vaccine, Recombinant MAGE-3.1 Antigen, MART-1 Antigen	Terminated		•Recurrent Melanoma •Stage IV skin melanoma	NCT01307618
recombinant IL-12	IFN-α	Completed	I	•Leukemia •Lymphoma •Multiple myeloma and plasma cell	NCT00003451
recombinant IL-12	IL-2	Completed	I	•Unspecified adult solid tumor, protocol specific	NCT00005604
recombinant IL-12	IL-2	Terminated	I	•Recurrent neuroblastoma	NCT00054405
recombinant IL-12	Recombinant IFN-α	Completed	II	•Recurrent melanoma •Stage IV melanoma	NCT00026143
recombinant IL-12	PSA prostate cancer vaccine	Completed	II	•Prostate cancer	NCT00015977
recombinant IL-12	recombinant IFN-α	Completed	I	•Kidney cancer •Melanoma (skin)	NCT00004244
rhIL-12	rhIL-2	Terminated	I	•Kidney neoplasm •Lung neoplasm •Sarcoma •Breast neoplasm	NCT00005655

## Conclusion and prospects

**Fig. 2:**
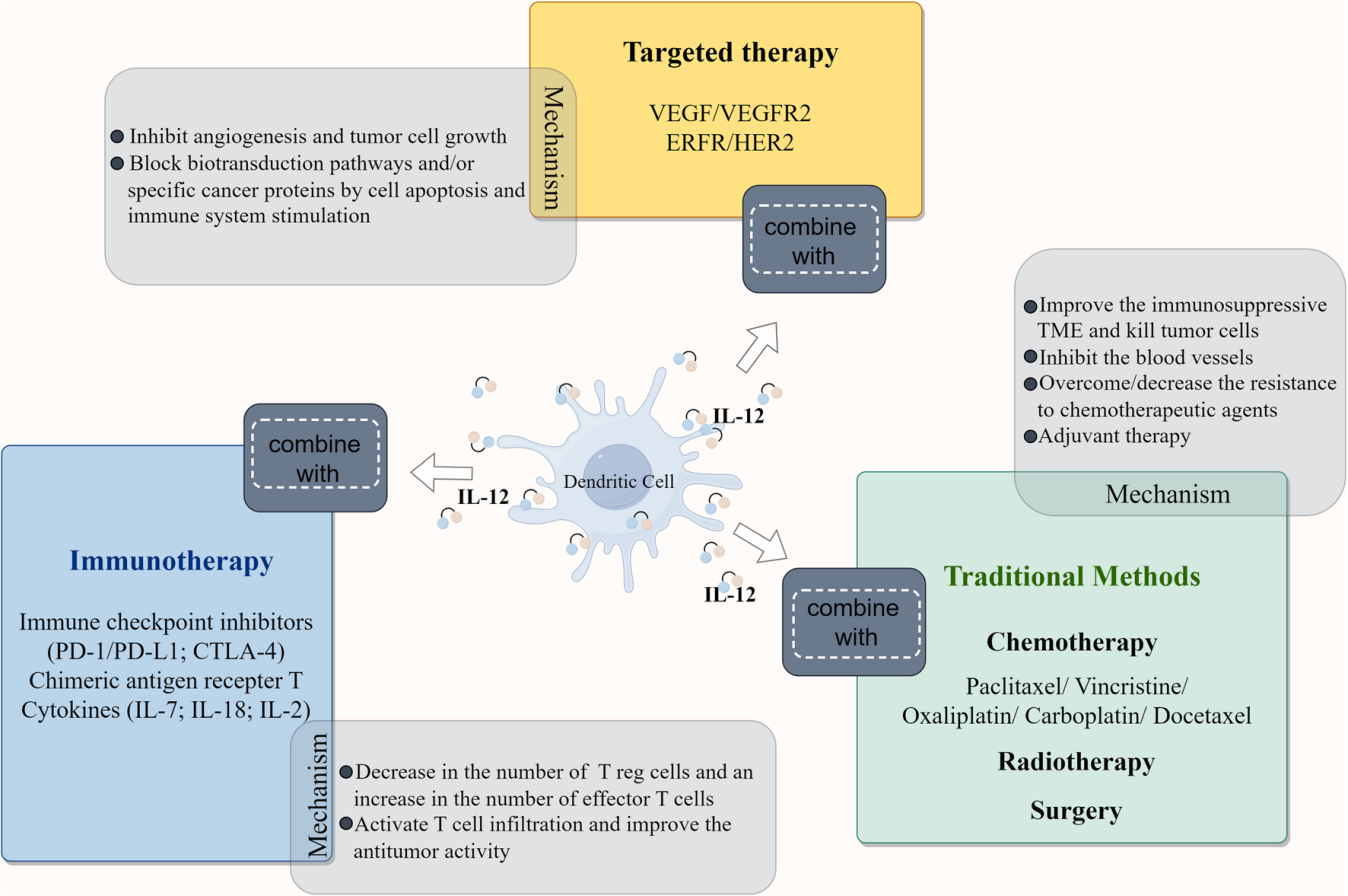
The summary of IL-12 combined with other therapies. Potential combination strategies include traditional methods (chemotherapy, radiotherapy and surgery), targeted therapy and immunotherapy, and the potential mechanisms involved in the combined treatment induce synergistic effects, such as inhibition of angiogenesis, regulation of T cell infiltration, activation of Treg cells, and so on

In summary, traditional methods (chemotherapy, radiotherapy and surgery), targeted therapy and immunotherapy have the potential to boost the anti-tumor effect of IL-12 and extend its beneficial effects in cancer patients. Figure 2 provides a graphical summary of the combination of IL-12 and other therapies, and shows some potential mechanisms. The combination of IL-12 and traditional methods, IL-12 can act as an adjuvant therapy to achieve the purpose of anti-tumor therapy by the anti-tumor effect or the immunoregulatory effects. When combined with chemotherapy, IL-12 can act additively or synergistically with chemotherapeutic agents in many studies. It has been demonstrated in many studies that the anti-tumor effect of IL-12 combined with chemotherapy is more significant than that of monotherapy. Specifically, this treatment strategy mainly showed on the killing of tumor cells and the inhibition of the blood vessels inside the tumor. Moreover, it can overcome/decrease resistance to chemotherapeutic agents. The combination of IL-12 and targeted therapies mainly focus on inhibition of angiogenesis or the growth of tumor. As for the radiotherapy and surgery, IL-12 always plays a role of adjuvant in the combination therapy by immunoregulatory effects. As for the combination of IL-12 with immunotherapies, such the most popular immune checkpoint inhibitor anti-PD-1/PD-L1. Combination treatments aimed at increasing the number and proportion of responding immune cells will be an effective treatment options for cancer patients. The combination of IL-12 and immunotherapy showed powerful anti-tumor effects in preclinical trials, but most of the relevant clinical trials are still in phase I. Therefore, there is still a long way to go for IL-12 combination therapy.

In the combination therapies, the explorations of delivery methods and tumor-targeted modification of IL-12 are also involved. Although IL-12 can effectively activate immune cells to enhance cytotoxicity and cell-mediated immune responses, its translation to the clinic has proven to be very difficult because of IFN-γ-associated toxicity. The strategies of local delivery and tumor targeting are also considered the potential approaches to mitigate the side effects currently. Local delivery strategies, mainly involving the delivery of IL-12-encoding nucleic acids directly into the tumor, are very promising. For example, the use of IL-12 for local injection improves the potential for local IL-12 delivery at the local tumor site, reduces systemic IL-12 exposure, and improves tolerance [100–103]. IL-12 expression was extremely localized, and plasma IL-12 levels were often undetectable in serum. Local delivery can maximize IL-12 delivery to the TME while minimizing systemic exposure, which have demonstrated robust antitumor immunity with reduced adverse events in preclinical studies. It may allow IL-12 to fulfill its considerable clinical potential.

As for the strategy of targeted tumors, most of them are to modify IL-12 itself, by linking tumor-binding antibody fragments or other targeting parts with IL-12, so as to promote the accumulation in tumors after systemic injection. The modification of IL-12 can be unfolded by the following points: (1) Targeting tumor antigens, which are overexpressed or uniquely expressed by tumor cells. Such as IL12-SS1, combined by IL-12 and scFv, can direct IL-12 to mesothelin expressing cancer cells, and it has been demonstrated in human peritoneal mesotheliomas established in nude mice [104]; (2) Targeting extracellular matrix epitopes found only in tumors. HuBC1-IL12 [105] has been developed to targeted the splice variant extra domain B (ED-B) of fibronectin, which is highly expressed in tumor tissues but few in normal adult tissues with the exception of endometrium; (3) Targeting tumor necrosis. NHS-IL12 is one of the most well-known fusion proteins, which consists of antibodies directed against histones of necrotic cells and functional domains of IL-12. And it has been verified in many experiments include combination treatment of IL-12 and other therapies [106–109]. Besides, researchers have adopted a variety of new strategies and methods, such as virus vectors, DNA delivery based on several polymers and lipids, DNA transfer by electroporation, etc. [110]. Notably, combination administration of IL-12 with other treatments in preclinical models showed in this review provides comparable or even better efficacy than single cytokines and offers the potential advantage of using lower and possibly better tolerated doses of IL-12 in clinical settings.

Collectively, previous studies have been demonstrated that IL-12 itself has powerful anti-tumor effects by itself from the studies performed to date and possesses a significant ability to synergize and cooperate with many other therapies (Table 3). The studies reported here enhance our understanding of the cellular and molecular mechanisms underlying the immunoregulatory and anti-tumor effects of these combination treatments. At present, it is difficult to predict which IL-12-based therapy will prevail in terms of safety and efficacy or which combination strategy will gain market acceptance. The results of these studies provide a basis for further clinical translation of IL-12-based combinatorial therapies for the treatment of various human cancers.

**Table 3:** Clinical trials of IL-12 combined with others

IL-12	Combination regimen	Status	Phase	Condition	CT number
M9241	ADT, Prednisone, M7824, Docetaxel	Recruiting	I and II	•Cancer of prostate •Prostate neoplasms	NCT04633252
GEN1	Paclitaxel, Carboplatin, Bevacizumab	Recruiting	II	•Ovarian cancer •Fallopian tube cancer •Primary peritoneal cancer	NCT05739981
IL-12	Dendritic cell/tumor fusion vaccine, Temozolomide	Recruiting	I and II	•Glioblastoma •Glioma •Neoplasms	NCT04388033
IL-12 gene therapy	Docetaxel, Pembrolizumab	Recruiting	II	•Triple Negative Breast	NCT04095689
Plasmid of IL-12	Pembrolizumab, nab paclitaxel	Recruiting	II	•Triple Negative Breast	NCT03567720
IL-12 gene therapy	Fludarabine, Cyclophosphamide, IL-12 transduced TIL	Terminated	I and II	•Skin cancer •Metastatic Melanoma	NCT01236573
rIL-12	MART-1 antigen, gp100 antigen, incomplete Freund’s adjuvant, sargramostim, tyrosinase peptide, alum adjuvant	Completed	II	•Intraocular melanoma •Melanoma (skin)	NCT00031733
rIL-12	Trastuzumab, paclitaxel	Completed	I	•Male breast cancer •Recurrent breast cancer •Recurrent gastric cancer	NCT00028535

## Data Availability

Not applicable.

## Abbreviations

AEs: Adverse events
Angio: Angiostatin
CAR-T: Chimeric antigen receptor T
CEA: Carcinoembryonic antigen
CR: Complete response
EGFR: Epidermal growth factor receptor
FDA: The US Food and Drug Administration
GBM: Glioblastoma
HER2: Human epidermal growth factor receptor 2
ICIs: Immune checkpoint inhibitors
IL-12: Interleukin-12
IL-18: Interleukin-18
IL-2: Interleukin-2
IL-7: Interleukin-7
IFN-α: Interferon-alfa
IL-12R: IL-12 receptor
mAngio: Mouse angiostatin
OR: Objective response
OXP: Oxaliplatin
PD-L1: Programmed cell death 1 ligand 1
PTX: Paclitaxel
SCCHN: Squamous cell carcinoma of the head and neck
Teff: Effector T cells
Th1: T helper 1 cells
TME: Tumor microenvironment
Tregs: Regulatory T cells
VB: Vincristine
VEGF: Vascular endothelial growth factor
VEGFR2: Vascular endothelial growth factor receptor 2
VEGF_121_: Vascular endothelial growth Factor subtype 121
